# Single Agent Polysaccharopeptide Delays Metastases and Improves Survival in Naturally Occurring Hemangiosarcoma

**DOI:** 10.1155/2012/384301

**Published:** 2012-09-05

**Authors:** Dorothy Cimino Brown, Jennifer Reetz

**Affiliations:** Veterinary Clinical Investigations Center, Department of Clinical Studies, School of Veterinary Medicine and University of Pennsylvania, 3900 Delancey Street, Philadelphia, PA 19104-6010, USA

## Abstract

The 2008 World Health Organization World Cancer Report describes global cancer incidence soaring with many patients living in countries that lack resources for cancer control. Alternative treatment strategies that can reduce the global disease burden at manageable costs must be developed. Polysaccharopeptide (PSP) is the bioactive agent from the mushroom *Coriolus versicolor*. Studies indicate PSP has in vitro antitumor activities and inhibits the growth of induced tumors in animal models. Clear evidence of clinically relevant benefits of PSP in cancer patients, however, is lacking. The investment of resources required to complete large-scale, randomized controlled trials of PSP in cancer patients is more easily justified if antitumor and survival benefits are documented in a complex animal model of a naturally occurring cancer that parallels human disease. Because of its high metastatic rate and vascular origin, canine hemangiosarcoma is used for investigations in antimetastatic and antiangiogenic therapies. In this double-blind randomized multidose pilot study, high-dose PSP significantly delayed the progression of metastases and afforded the longest survival times reported in canine hemangiosarcoma. These data suggest that, for those cancer patients for whom advanced treatments are not accessible, PSP as a single agent might offer significant improvements in morbidity and mortality.

## 1. Introduction

The 2008 World Health Organization World Cancer Report describes the global cancer incidence soaring, with 20 million new patients each year by 2020 [1]. A large percentage of them will live in countries that lack the resources for cancer control. The dramatic technological changes that will continue in surgery, radiotherapy, and chemotherapy will lead to increased cure rates; however, these anticipated advances come at a price usually beyond the means of most cancer patients. It is imperative that affordable complementary and alternative treatment strategies, that could considerably reduce the global disease burden at manageable costs, are developed [2]. 

The role of complementary and alternative medicine continues to evolve in the treatment regimen of cancer patients. Herbal medicines have predominantly been regarded for promoting wellness through the potentiation of immune functions. Beginning in the 1990s however, it has become increasingly clear that mushrooms and mushroom extracts have activities beyond that of the immune system. They have the potential to directly suppress tumorigenesis [3]. 

The mushroom *Coriolus versicolor*, known in China as Yun Zhi, has been used in herbal medicine for more than 2000 years. The bioactive agent in the mushroom is believed to be a polysaccharopeptide (PSP) [2]. Pharmacological studies indicate that PSP has in vitro antitumor activities, while having little inhibitory effect on normal cell lines. In laboratory animal experiments, PSP has been shown to inhibit the growth of induced tumors, without measurable toxicity [4–6]. As with all other preclinical data, whether these reported findings from the rodent models have relevance in patient care is uncertain [7, 8], but it seems possible that this dietary supplement could offer beneficial effects on survival and quality of life in cancer patients [9]. Clear evidence of clinically relevant benefits of PSP in cancer patients, however, is lacking.

Randomized controlled trials investigating the efficacy of PSP in cancer patients report positive results for markers of immune function but lack clear evidence for antitumor activity and the ultimate clinically relevant outcome of increased survival [10–14]. These trials are few in number, have small sample sizes, are of short duration, and are often confounded by comorbidities as well as concurrent therapies. The investment of the sizable resources required to complete large-scale, long-term, and randomized controlled trials of PSP in cancer patients would be more easily justified if antitumor and survival benefits could be documented in a complex animal model of a naturally occurring cancer that parallels the human disease. Hemangiosarcoma in companion dogs is such a model.

Because of its high metastatic rate and vascular origin, canine hemangiosarcoma has significant model potential for investigations in antimetastatic therapies in general and antiangiogenic modalities in particular [15–18]. It is a malignant neoplasm of vascular origin that occurs in middle-aged to older dogs. The most common primary site is the spleen and it is typified by very aggressive biologic behavior with rapid and widespread metastases. Metastasis is typically hematogenous or through transabdominal implantation following spontaneous rupture of the splenic mass. Overall, the prognosis for dogs treated by splenectomy alone is extremely poor; median survival times range from 19 to 86 days, and less than 10% survive to 12 months [19–22]. The primary objective of this pilot study was to determine if dose-dependent antitumor and survival effects of single agent Yun-Zhi PSP (I'm-Yunity) could be documented in naturally occurring canine hemangiosarcoma, in which the cohort is randomized to dose, investigators are blinded to dose, and concurrent therapies are not administered.

## 2. Materials and Methods

### 2.1. Study Design and Overview

This pilot study was a randomized, double-blind, and multidose study of I'm-Yunity in dogs with a histopathologic diagnosis of splenic hemangiosarcoma. Dogs were recruited from a university-based tertiary care veterinary academic teaching hospital. A computer generated randomization sequence with 3 potential treatment groups (I'm-Yunity 25, 50, or 100 mg/kg/day) was generated at an off-site pharmacy. The randomization scheme was stratified according to the presence or absence of metastatic disease documented at the time of diagnosis. The sequence was concealed so that no members of the research team were aware of the group to which a dog would be allocated as it was evaluated in the screening process. Once screening was completed and a dog was considered eligible for inclusion in the study, a unique study number was assigned in sequence. The study number and body weight of each dog were then provided to the pharmacy. Pharmacy personnel matched the study number with their randomization sequence, formulated the appropriate treatment, and packaged capsules for distribution by the investigators. Capsules were formulated to appear identical. Thus, all study personnel and the owners of the dogs were unaware of the dose group to which each dog was assigned. Owners administered the capsules to their dogs daily as prescribed until the death of the dog. In-hospital evaluations of the dogs occurred at screening, baseline, and at monthly intervals until each dog's death. Each dog underwent a full necropsy following its death. 

### 2.2. I'm-Yunity

I'm-Yunity (brand name) is an extract of *Coriolus versicolor* mushroom commonly referred to as cloud mushroom, turkey tail, or Yunzhi mushroom in China.The mushroom's active ingredient, to which the product is standardized, is PSP, which is short for polysaccharopeptide or polysaccharide peptide (both names are used in the literature). It is important to mention brand namewhen stating PSP use in this study because various brands can have varying amounts of polysaccharides and peptides in what they claim is PSP. In the literature, it is commonly refereed to just as PSP; however, all PSP's may not be the same as it is considered a general term for a combination of polysaccharides and peptides together and potentially PSP could be isolated from another mushroom altogether [23, 24]. I'm-Yunity is *Coriolus versicolor* mushroom extract, specifically from the cov-1 strain. PSP in the form of I'm-Yunity is isolated from the mycelium of the mushroom and not the fruiting body as is the claim with other brands. It was supplied by Integrated Chinese Medicine Holdings, Ltd. (Hong Kong, China), as a mushroom product produced, using a proprietary fractionation scheme, from deep-layer cultivated mycelia of cov-1 according to good manufacturing practice (GMP) standards. The molar ratio of monosaccharides in polysaccharide moiety of I'm-Yunity PSP is glucose : galactose : mannose : xylose : arabinose = 8.2 : 1.5 : 2.4 : 2.4 : 1.0.In I'm-Yunity PSP, soluble polysaccharideis 46%, and soluble proteinis 31%. The amino acids in the protein portion are presented in Table 1 [25]. Quality control of I'm-Yunity involved determination of heavy metal contents, microorganism contamination, authentication of elution properties on high pressure liquid chromatography, and supplementary bioactivity-guided assays, performed by laboratories at the manufacturing facilities and also independently by commercial testing centers.

### 2.3. Eligibility

Dogs were screened for enrollment from September 2008 through November 2009 following Institutional Animal Care and Use Committee approval by the University and written informed consent by the owners of the dogs. Inclusion criteria were histopathologic diagnosis of splenic hemangiosarcoma and life expectancy ≥4 weeks. Only dogs whose owners opted not to pursue chemotherapy following splenectomy were eligible for enrollment.

### 2.4. Evaluation of Response

All dogs were evaluated at baseline to ensure that they were stable in their general condition. Hematological and biochemical tests (complete blood count, electrolytes, liver and renal function), 3-view digital thoracic radiographs, and contrast enhanced harmonic abdominal ultrasound [26] for baseline assessment of abdominal metastatic disease (liver, omentum, mesentery) were performed. All diagnostics were repeated at monthly follow-up visits. Reports, digital images, and video recordings of the baseline studies were compared to each monthly follow-up study. Based on this comparison, the board certified radiologist made the determination of progression of metastatic disease. Time to progression was defined as the number of days from the baseline assessment to the first evaluation day in which progression was documented by the radiologist based on contrast enhanced harmonic abdominal ultrasound. 

### 2.5. Statistical Analysis

Descriptive statistics were calculated. Continuous data were expressed as mean and SD, unless not normally distributed, in which case median values and ranges were reported. Categorical data were expressed as frequencies. Because of the nonnormality of the data, the Kruskal Wallis test was used to evaluate the changes that occurred in complete blood count and serum biochemistry parameters within each dose group and between dose groups over time. The Mann Whitney test was used to compare the median days to progression of abdominal metastases between the high-(100 mg/kg/day) and low-(25 mg/kg/day) dose groups. Median survival times were determined by the use of the Kaplan-Meier product limit method and log rank analysis was used to compare survival curves amongst dose groups. *P* < 0.05 was considered statistically significant and all analyses were performed using STATA 11.

## 3. Results

The baseline characteristics of 15 dogs with splenic hemangiosarcoma enrolled in the study were equally distributed amongst the three treatment groups (25 mg/kg/day, 50 mg/kg/day, or 100 mg/kg/day) via randomization and are presented in Table 2. In a double-blind (investigator and owner) manner, all dogs received capsules containing PSP daily from baseline assessment until death. One dog, in the 25 mg/kg/day dose group, developed hypercalcemia secondary to a parathyroid adenoma eight months after enrolling in the study. This dog was maintained on I'm-Yunity throughout treatment for the adenoma and was followed with all evaluations according to protocol until death. No unexpected comorbidities developed in any of the other dogs. There was no significant variability in any complete blood count or serum biochemistry parameter in any dose group or between dose groups through the course of the study (Tables 3 and 4).

### 3.1. High-Dose PSP Delayed the Progression of Metastases

**Figure 1:**
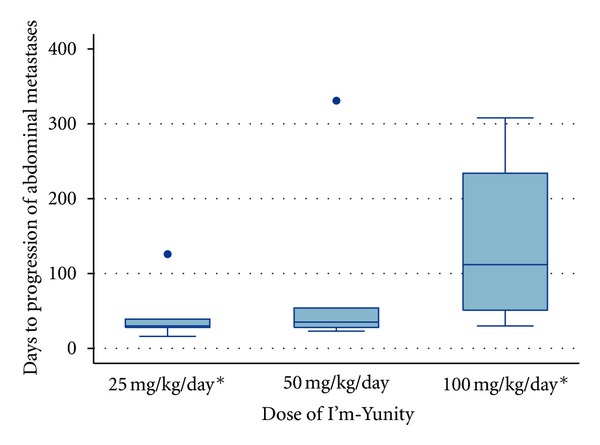
Days to progression of abdominal metastases for 15 dogs diagnosed with splenic hemangiosarcoma randomized to one of three doses of I'm-Yunity (5 dogs in each dose group). ∗ Denotes a statistically significant difference (*P* = 0.046) in median days to progression of abdominal metastases between groups (30 versus 112 days).

The median time to development or progression of abdominal metastases was significantly delayed in dogs receiving 100 mg/kg/day I'm-Yunity (112 days; range 30 to 308 days) compared to dogs receiving 25 mg/kg/day (30 days; range 16 to 126 days; *P* = 0.046). Dogs receiving 50 mg/kg/day I'm-Yunity had median 35 days to progression of metastases (range 23 to 331 days), which was not significantly different than the 25 or 100 mg/kg/day groups (Figure 1).

### 3.2. Median Survival Time Increased as Dose of PSP Increased and High Dose PSP Provided the Longest Median Survival Times Reported to Date

**Figure 2:**
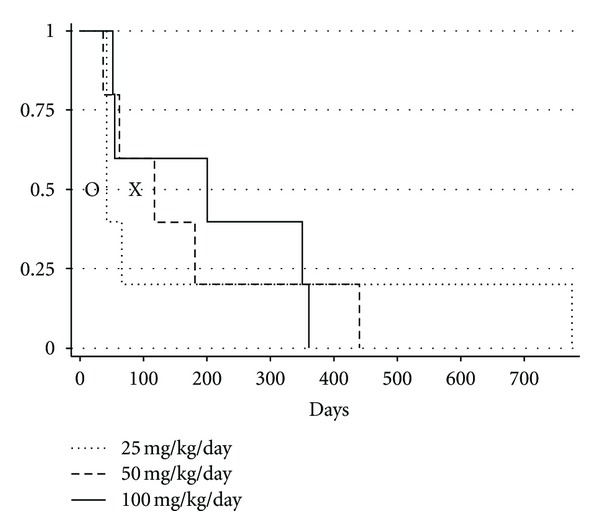
Kaplan Meier survival curves for 15 dogs diagnosed with splenic hemangiosarcoma randomized to one of three doses of I'm-Yunity (5 dogs in each dose group). “O” represents the shortest median survival time reported in the literature (19 days). “X” represents the longest median survival time reported in the literature (86 days).

The Kaplan Meier survival curves for the three dose groups are presented in Figure 2. While there was no statistically significant difference in the survival curves amongst the 3 dose groups, the two highest dose groups had median survival times longer than the longest median survival time reported in the literature to date, which is 86 days, as compared to 117 days in dogs receiving 50 mg/kg/day I'm-Yunity and 199 days in dogs receiving 100 mg/kg/day.

## 4. Discussion

The goal of this study was to collect pilot data on the potential effect of I'm-Yunity on the morbidity (progression of abdominal metastases) and mortality associated with a naturally occurring cancer in a large and complex species. The study was designed to collect outcome data so that a definitive proof-of-concept study could be appropriately powered, while also evaluating a range of potential doses. The goals of the study were met as expected; however, the magnitude of the positive outcomes and their relationship to dose was unexpected in such a small cohort, particularly in such an aggressive neoplastic disease.

**Figure 3:**
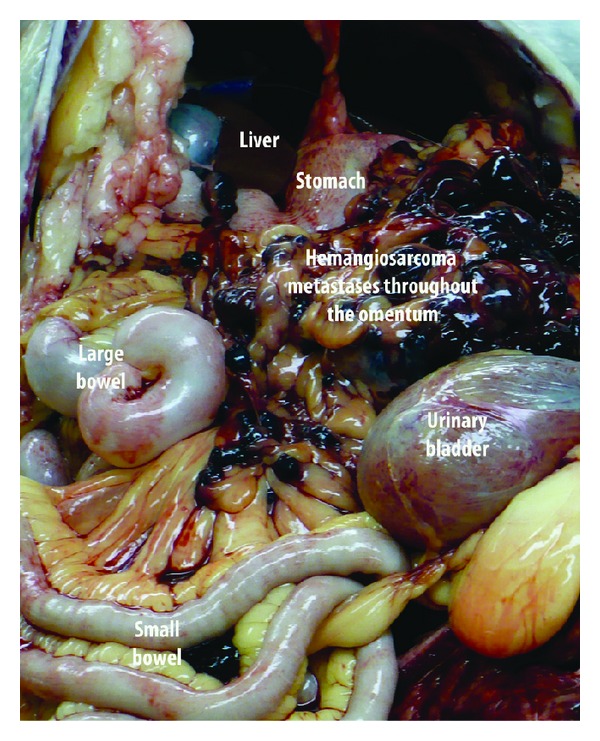
Metastatic hemangiosarcoma throughout the omentum at necropsy of a dog with splenic hemangiosarcoma 6 months following splenectomy.

Dogs with splenic hemangiosarcoma are often asymptomatic until the tumor ruptures causing the dog to collapse due to hemorrhagic/hypotensive shock. Many owners will opt for emergency surgical removal of the spleen to save the dog's life through the crisis, despite the fact that the long-term prognosis for the dog could be very poor since 70% of dogs with splenic masses presenting with nontraumatic hemoabdomen do indeed have hemangiosarcoma as the underlying pathology [27]. Despite the fact that metastatic disease may not be identified at the time of splenectomy, rapid and widespread metastatic disease to the liver, omentum and/or mesentery, will typically lead to the development of a life-ending hemorrhagic shock crisis within 3 months of the splenectomy (Figure 3). Given the high metastatic rate and poor outcome associated with surgery alone, adjuvant chemotherapy using single agent and combination doxorubicin based protocols can be used, with reported median survival times of 141 to 179 days [28–30]. Because chemotherapy is costly, time intensive, and does not increase the 12 month survival rate above that seen with surgery alone, most owners do not opt for chemotherapy. The goal for these dogs is to maintain their quality of life for the final months of their life. It is this cohort of dogs that are ideal for enrolling in proof-of-concept studies for therapies that have some evidence for antitumor effects.

I'm-Yunity has demonstrated antitumor activities in tissue culture studies, based principally on data using flow cytometry. It causes cell cycle arrest and alterations in the expression of apoptogenic/antiapoptotic and extracellular signaling proteins, the net result being a reduction in proliferation and an increase in apoptosis [3]. Dose-dependent, time-dependent growth suppression, and decrease in cell viability have been documented for HL-60 leukemic cells incubated with I'm-Yunity [2]. I'm-Yunity appears to act selectively in HL-60, resulting in cell cycle restriction through the G1/S checkpoint and the induction of apoptosis. PSP has also been shown to inhibit growth of human hepatoma cell lines [31]. In animal models, PSP inhibits tumor growth in sarcoma 180 inoculated nude mice and slows progression in a herpes virus type 2-transformed murine tumor (H238) model [32]. As with all other preclinical data, whether these reported findings of suppression of cell proliferation and induction of apoptosis in malignant cells from murine models have relevance in patient care is uncertain [9]. 

PSP has been widely used as therapeutic adjuvant for cancer immunotherapy in China and Japan [33–36]. In phases I and II of double blind trials in China, the use of PSP has been shown to boost immune cell proliferation, alleviate chemotherapy symptoms, and enhance tumor infiltration by dendritic cells and cytotoxic T lymphocytes [37]. In a recent clinical trial, PSP treatment increased blood leucocyte and neutrophils counts, as well as serum IgG and IgM concentrations in patients with advanced nonsmall cell lung cancer [38]. Administration of PSP to esophageal cancer, gastric cancer, or lung cancer patients with radiotherapy or chemotherapy can alleviate symptoms and prevent the decline of immune status [34]. There were no significant changes in complete blood count and serum chemistry parameters in any of the 3 dosage groups over time. The majority of animals in all three groups had baseline white blood cell and serum chemistry parameters very close to, or in, the normal range and maintained those numbers throughout the study. One could speculate that PSP played a role in maintaining the “health” of these parameters; however, a negative control group would be needed to support such a claim. As would be expected, many dogs entered the study with red blood cell parameters that reflected the recent bleeding episode that necessitated splenectomy. There were no statistically significant improvements in the value of these parameters over time, which is due to intermittent bleeding episodes associated with the metastatic disease that developes in the omentum, mesentery, and liver. While randomized controlled trials investigating the efficacy of PSP in cancer patients report these positive results for markers of immune function and possible mitigation of chemotherapeutic or radiation treatment side effects, they lack clear evidence for antitumor activity and the ultimate clinically relevant outcome of increased survival [10]. 

There could be several reasons for the lack of clear evidence of the clinically relevant antitumor effects in clinical trials, the first of which is, perhaps there are no such positive effects associated with PSP in cancer patients to be identified. The inability of preclinical findings to predict clinical efficacy is a concern throughout biomedical research and must be considered as a possible reason for the disconnect between preclinical and clinical data to date [7, 8]. A second reason could be that the clinical trials are not designed with the best chance to identify a positive result for the clinically relevant outcomes of tumor progression and survival. There are very few controlled clinical trials and they generally have small sample sizes and are not powered to detect a difference in tumor progression or survival. They are often of short duration, with PSP administered for a relatively limited period of time (weeks). In addition, the data in these trials can be confounded by comorbidities associated with patients in advanced stages of disease in most of these trials. A third reason that it may be difficult to identify a positive effect of PSP on tumor progression and survival in reported clinical trials is that, in nearly all studies, patients are receiving or have received standard of care chemo- or radiation therapy. It is possible that PSP has an antitumor effect “as good as” chemo- or radiation therapy, but that effect is masked by the presence of those interventions in both the treatment and control arms of the trial. Ultimately, the only way to definitively determine whether PSP can delay tumor progression and improve survival in cancer patients is for large-scale, long-term, and randomized controlled trials to be performed. The investment of the very sizable resources required to complete such trials could be more easily justified if positive results in the clinically relevant outcome measures that are of importance in cancer patients were clearly documented in an animal model. 

The use of animals as models to address preclinical study of cancer therapeutics has a long history, and important information has been acquired on new and innovative therapies. Most of these investigations have used inbred rodent models and laboratory-derived canine populations. Working with inbred populations in laboratory environments raises some degree of concern over the applicability of information as it relates to naturally occurring tumors in people. While the choice of companion dog model for this proof-of-concept pilot data collection is outside the traditional laboratory animal paradigm, it offers some interesting and useful advantages.

The degree of medical surveillance of the dog is second only to that in humans. The dog's state of health is observed in intimate detail on a day-to-day basis and its ailments are attended by veterinary specialists using all of the diagnostic approaches of modern medicine [39]. Dogs develop cancer about twice as frequently as humans, and the presentation, histology, and biology of many canine cancers closely parallel those of human cancers [15, 39]. Dogs share the environment with people and thus also share the potential environmental risk factors for disease and have comparable responses to treatment with cytotoxic agents. The large body size simplifies biologic sampling and the shorter overall lifespan of the dog allows for spontaneous development and time course of disease within a time frame reasonable for efficient data collection. In the case of hemangiosarcoma, a highly metastatic spontaneous malignancy arising most often in the spleen, most dogs die within 3 months of diagnosis due to metastatic disease [19, 21, 22, 40]. This is a time period long enough to accumulate meaningful clinically relevant morbidity and mortality information, but short enough to be efficient in the use of investigative resources. The value of the canine hemangiosarcoma model of disease goes well beyond investigations into hemangiosarcoma as a primary neoplasm. Its value in impacting human health lays in its high metastatic rate and vascular origin, which makes it uniquely suited for investigations in antimetastatic therapies in general and antiangiogenic modalities in particular [15–18]. This held true for the objectives of this study.

It was not anticipated that such clear dose-dependent patterns in efficacy data would emerge or that any statistically significant dose-related information would be identified in such a small cohort. While it is necessary to now document more robust and statistically significant differences in an appropriately powered definitive placebo controlled study, it is encouraging that clear patterns and an obvious dose choice emerged from the pilot program. The median time to development or progression of abdominal metastases was significantly delayed in dogs receiving 100 mg/kg/day I'm-Yunity, which was documented on enhanced harmonic abdominal ultrasound. It was also reflected in the fact that while the majority of dogs never developed a normal hematocrit because of low grade bleeding episodes from abdominal metastases, four dogs did achieve a normal hematocrit during the course of the study and all four dogs were in the 100 mg/kg/day dose group. In addition, dogs in this dose group had the longest median survival times reported to date. The fact that these animals had not received adjuvant therapies such as chemotherapy in addition to the PSP, may have allowed the antitumor effects of PSP to be more readily realized. It is noteworthy that the median survival time for dogs in the 100 mg/kg/day treatment group (199 days) was longer than that which is reported for dogs receiving doxorubicin based chemotherapeutic protocols (141 to 179 days). Based on this data, one could hypothesize that PSP has the potential to have effects on survival similar to that which is seen with standard of care chemotherapy. Proving, in a biologically aggressive animal model, that PSP delivers antitumor and survival effects in a magnitude similar to that which is seen in standard chemotherapy could have significant implications for shifts in standard of care from current cytotoxic therapies to complementary compounds, such as PSP, that have little to no negative documented effects on normal cells. Most importantly, for those cancer patients throughout the world for whom advanced treatments and cytotoxic therapies are not accessible, CAM, such as PSP as a single agent, could offer benefits to survival and quality of life that are not yet imagined for those populations.

## Figures and Tables

**Table 1:** Amino acids in the protein portion of I'm-Yunity PSP (polysaccharopeptide).

Amino acid	Content (%)
Aspartic acid	4.0
Threonine	2.3
Serine	3.2
Glutamic acid	5.8
Proline	1.0
Glycine	2.6
Alanine	2.6
Cysteine	0.9
Valine	1.8
Methionine	0.4
Isoleucine	2.2
Leucine	2.4
Tyrosine	1.5
Phenylalanine	1.5
Tryptophan	1.7
Lysine	2.3
Histidine	0.7
Arginine	1.8

**Table 2:** Baseline characteristics of 15 dogs diagnosed with splenic hemangiosarcoma randomized to one of three doses of I'm-Yunity.

	I'm-Yunity (polysaccharopeptide)		
	25 mg/kg/day	50 mg/kg/day	100 mg/kg/day
	(*n* = 5)	(*n* = 5)	(*n* = 5)
Breed	1 mixed breed (20%)	2 mixed breed (40%)	2 mixed breed (40%)
	4 pure breed (80%)	3 pure breed (60%)	3 pure breed (60%)
Age (years)	9.0 ± 1.9	9.6 ± 2.7	8.6 ± 1.1
Weight (kg)	34.0 ± 7.0	28.2 ± 4.8	30.0 ± 12.8
Sex	1 female (20%)	2 female (40%)	1 female (20%)
	4 male (80%)	3 male (60%)	4 male (80%)
Metastatic disease present at enrollment	2 no (40%)	2 no (40%)	2 no (40%)
	3 yes (60%)	3 yes (60%)	3 yes (60%)
Time from diagnosis to study enrollment (days)	16 ± 9	23 ± 10	20 ± 8

**Table 3:** Median and range blood chemistry values for 15 dogs with hemangiosarcoma treated with 25, 50, or 100 mg/kg/day I'm-Yunity (polysaccharopeptide).

Normal values	25 mg/kg/day			50 mg/kg/day			100 mg/kg/dy		
	Baseline (*n* = 5)	Week 3 (*n* = 4)	Week 7 (*n* = 1)	Baseline (*n* = 5)	Week 3 (*n* = 5)	Week 7 (*n* = 2)	Baseline (*n* = 5)	Week 3 (*n* = 5)	Week 7 (*n* = 3)
Glucose 65–112 mg/dl	79 (73–122)	88 (77–106)	91	101 (87–121)	100 (84–108)	100 (96–103)	87 (75–101)	96 (86–126)	91 (90–92)
BUN 5–30 mg/dl	17 (10–26)	25 (13–31)	27	18 (17–23)	19 (15–25)	19 (18–20)	25 (18–55)	27 (20–34)	26 (17–39)
Creatinine 0.7–1.8 mg/dl	0.9 (0.7–1.5)	1.4 (0.6–1.8)	1.9	1.0 (0.9–1.3)	1.0 (0.9–1.5)	1.0 (0.8–1.2)	1.2 (0.8–1.5)	1.2 (1.1–1.2)	1.0 (0.9–1.3)
Calcium 9.8–11.7 mg/dl	10.4 (9.7–12.0)	10.3 (9.1–12.0)	12.0	9.7 (9.6–11.0)	10.2 (9.8–10.8)	10.0 (9.6–10.3)	10.2 (9.6–10.7)	10.2 (9.1–11.4)	10.1 (10.1–10.7)
Sodium 140–150 nmol/L	144 (132–148)	141 (138–146)	141	146 (139–147)	144 (143–147)	143 (142–143)	146 (143–146)	146 (140–152)	146 (143–146)
Potassium 4.0–5.2 nmol/L	4.9 (4.6–5.4)	4.4 (3.4–5.1)	5.1	4.6 (4.4–5.6)	4.5 (3.7–4.6)	4.4 (4.1–4.7)	4.8 (4.0–5.0)	4.8 (3.5–5.6)	4.6 (4.5–5.1)
Total protein 5.4–7.1 g/dl	6.3 (5.6–6.7)	6.0 (4.4–7.0)	5.5	6.0 (5.3–7.0)	5.9 (5.6–6.3)	5.8 (5.7–5.9)	5.9 (5.7–6.4)	6.1 (4.8–6.5)	6.4 (6.3–6.5)
Albumen 2.5–3.7 g/dl	3.0 (2.8–3.3)	3.0 (2.2–3.8)	3.2	3.2 (3.0–4.0)	3.1 (2.9–3.5)	3.3 (3.2–3.4)	3.0 (2.9–3.5)	3.0 (2.5–3.4)	3.2 (3.1–3.4)
Globulin 2.4–4.0 g/dl	3.2 (2.8–3.6)	3.0 (2.2–3.2)	2.3	2.8 (2.2–3.4)	2.7 (2.5–3.1)	2.5 (2.3–2.7)	2.8 (2.3–3.3)	3.1 (2.1–3.2)	3.1 (3.1–3.3)
ALT 16–91 U/L	32 (13–57)	46 (20–89)	32	60 (13–102)	49 (27–64)	89 (33–145)	59 (14–159)	62 (46–118)	36 (26–60)
SAP 20–155 U/L	143 (59–346)	148 (55–575)	105	83 (29–828)	79 (41–688)	461 (49–872)	112 (37–156)	82 (47–184)	131 (93–166)
Total bilirubin 0.1–0.5 mg/dl	0.1 (0.1–0.3)	0.2 (0.1–0.2)	0.5	0.3 (0.1–0.9)	0.1 (0.1–0.2)	0.3 (0.2–0.3)	0.1 (0.1–0.5)	0.2 (0.1–0.3)	0.2 (0.2–0.3)
Cholesterol 128–317 mg/dl	260 (161–292)	300 (138–393)	343	247 (181–258)	233 (186–274)	242 (156–328)	220 (196–276)	260 (165–492)	332 (285–442)

**Table 4:** Median and range complete blood count values for 15 dogs with hemangiosarcoma treated with 25, 50, or 100 mg/kg/day I'm-Yunity (polysaccharopeptide).

Normal values	25 mg/kg/day			50 mg/kg/day			100 mg/kg/dy		
	Baseline (*n* = 5)	Week 3 (*n* = 4)	Week 7 (*n* = 1)	Baseline (*n* = 5)	Week 3 (*n* = 5)	Week 7 (*n* = 2)	Baseline (*n* = 5)	Week 3 (*n* = 5)	Week 7 (*n* = 3)
Red blood cells 5.9–8.9 × 10^6^ /*μ*l	5.3 (4.8–6.2)	4.3 (3.1–5.7)	4.3	5.8 (5.5–6.4)	6.0 (4.7–6.4)	4.2 (3.5–5.0)	5.2 (5.0–5.6)	5.7 (2.0–6.0)	5.4 (5.3–5.8)
Hemoglobin 13.3–20.5 g/dl	12.6 (11.4–14.4)	10.4 (7.0–13.4)	10.8	12.9 (12.2–14.3)	13.1 (10.3–14.3)	8.9 (7.2–10.7)	12.7 (11.8–17.0)	14.3 (4.7–14.9)	13.7 (13.4–14.6)
Hematocrit 40.3–60.3 %	36.2 (32.9–40.3)	29.4 (20.7–39.7)	30.8	37.1 (35.0–39.4)	37.5 (30.0–40.1)	26.2 (21.2–31.2)	35.4 (34.3–39.4)	40.7 (13.7–42.1)	37.8 (37.2–41.0)
Platelets 177–398 × 10^3^/*μ*l	639 (363–785)	422 (107–642)	536	441 (120–682)	272 (122–386)	117 (116–117)	577 (180–746)	569 (92.1–640)	532 (491–625)
White blood cell 5.3–19.8 × 10^3^/*μ*l	10.2 (9.1–12.5)	7.7 (5.6–29.4)	9.3	8.2 (7.0–15.8)	10.6 (6.9–13.3)	12.4 (8.1–16.7)	9.7 (6.9–11.8)	11.0 (8.3–23.2)	9.9 (9.7–13.2)
Neutrophils 3.1–14.4 × 10^3^/*μ*l	6.7 (6.1–8.6)	5.6 (4.3–26.2)	7.9	6.5 (4.8–14.1)	7.7 (4.5–12.0)	10.8 (6.5–15.0)	6.5 (5.0–10.2)	9.2 (6.2–20.0)	7.4 (7.2–9.2)
Lymphocytes 0.9–5.5 × 10^3^/*μ*l	1.9 (0.7–2.8)	1.0 (0.8–1.2)	0.8	0.8 (0.5–1.3)	0.7 (0.1–1.8)	0.7 (0.3–1.1)	0.9 (0.6–1.7)	1.7 (0.3–2.2)	2.0 (1.8–2.6)
Monocytes 0.1–1.4 × 10^3^/*μ*l	0.7 (0.5–1.3)	0.5 (0.3–2.1)	0.4	0.6 (0.4–1.0)	0.7 (0.3–1.4)	0.7 (0.5–1.0)	0.5 (0.1–0.7)	0.4 (0.3–0.7)	0.5 (0.2–0.8)
Eosinophils 0.0–1.6 × 10^3^ /*μ*l	0.7 (0.2–1.3)	0.4 (0.1–0.9)	0.2	0.3 (0.2–1.4)	0.5 (0.1–0.8)	0.1 (0.1–0.2)	0.4 (0.2–0.8)	0.5 (0.3–0.6)	0.3 (0.2–0.5)
